# Beyond proximity: The social and spatial dynamics of collaboration in community energy initiatives

**DOI:** 10.1111/bjso.70121

**Published:** 2026-08-07

**Authors:** Charlie J. Walker Clarke, Elliot J. Sharpe, Lise Jans, Thijs Bouman

**Affiliations:** ^1^ Faculty of Behavioural and Social Sciences University of Groningen Groningen The Netherlands

**Keywords:** collaboration and community energy, in‐group identification, interpersonal contact, perceived distance, spatial distance

## Abstract

This research examines how actual and perceived spatial distance from group members shape collaboration intentions in the context of community energy initiatives. While previous studies highlight in‐group identification and interpersonal contact for community engagement, the role distance plays in this has received less attention. Across two studies (N total = 195, N total = 287), we tested across spatially different groups (neighbourhood, city, nation, family, workplace) whether indicators of greater (perceived) spatial distance were indicative of less interpersonal contact, lower identification with the group and weaker collaboration intentions. Our results indicate that perceived spatial distance did not correspond to actual spatial differences across groups, suggesting that perceived distance does not always reflect actual distance. However, members who perceive other members as less distant report stronger interpersonal contact, identification (Studies 1 and 2) and collaboration intentions (Study 2). Associations between interpersonal contact, identification and collaboration intentions were consistent across groups, suggesting that these processes are rather universal, while the role and interpretation of distance fluctuate between the groups tested. These results contribute to t/0258heory by clarifying the interplay between spatial and social dimensions of groups, and by showing how perceptions of space shape identification and intentions to engage in community‐based energy.

## INTRODUCTION

Effective collaboration between individuals is a crucial aspect of pushing forward societal goals, such as the energy transition. Indeed, community energy initiatives are increasingly recognised as vital contributors to the energy transition (Schwanitz et al., 2023) and strongly rely on collaborations among members. Energy community initiatives broadly involve communities collectively promoting sustainable energy sources within a defined area. However, because their purposes, structures and processes vary widely, they lack a single, universally accepted definition (Bauwens et al., 2022). Crucially, however, these initiatives rely on the active participation and effective collaboration of individuals, who contribute time and resources through membership fees, meeting attendance and financial investments in managing and governing renewable electricity production, trade and storage within their communities (De Simone et al., 2025). One central factor in sustaining collaboration is in‐group identification (Bauwens, 2016), which reflects the extent to which individuals align themselves with their group or community.

Social psychological research shows that stronger in‐group identification facilitates collaborative behaviours, including collaboration and participation within the context of community energy initiatives (Goedkoop, Sloot, et al., 2022; Heath et al., 2017; Obst & White, 2007; Rees & Bamberg, 2014; Sloot et al., 2018). On top of this, interpersonal contact among group members is crucial for fostering a sense of belonging and strengthening identification with the group (Blanchard et al., 2020; Campbell, 1958; Jans et al., 2012, 2015; Postmes et al., 2005), while also directly predicting collaboration (Goedkoop, Sloot, et al., 2022; Thomas et al., 2016). This paper adopts a novel perspective by exploring how (perceived) spatial distance from group members shapes these social‐psychological processes. This is particularly relevant in the context of energy communities as these initiatives substantially vary in spatial composition and increasingly move away from the traditional neighbourhood‐level initiative. Specifically, we test the concept that less (perceived) distance to other group members strengthens social ties and, thereby, facilitates collaboration intentions among members. In doing so, we offer new insight into the conditions that encourage interpersonal contact, strengthen identification and may ultimately deepen collaboration intentions in community‐led energy initiatives.

This research investigates how both actual and perceived spatial distance from other in‐group members influence interpersonal contact, in‐group identification and collaborative intentions. Specifically, it examines whether greater (perceived) spatial distance between in‐group members acts as a limiting factor (Nientimp et al., 2024) that reduces interpersonal contact, in‐group identification and thus individuals' intentions to collaborate. In this research, we examine two core spatial characteristics. Study 1 investigates whether members of in‐groups at more local spatial scales (neighbourhood vs. city vs. national level) are perceived as more proximate, and whether this is associated with stronger interpersonal contact and in‐group identification. Study 2 focuses on different groups of comparable scale (i.e. neighbourhood, colleagues, extended family), examining instead how one's perceived spatial distance from other group members (i.e. centrality and density) may affect interpersonal contact, in‐group identification and collaboration intentions. Through this, we try to develop a deeper understanding of the interplay between spatial and social factors that shape people's intentions to collaborate in community‐based energy initiatives.

### Interpersonal contact and in‐group identification in fostering collaboration

In‐group identification is a key predictor of people's willingness to collaborate with those with whom they share a social identity (Haslam & Ellemers, 2011; Turner, 1991). Stronger in‐group identification has been found to increase individuals' motivation to engage in collaborative efforts that benefit the in‐group (Argo, 2020; Barth et al., 2021; Ellemers et al., 2002; Fritsche et al., 2018; Masson & Fritsche, 2021; Spears, 2025; Turner, 1991). This dynamic is particularly evident in research on energy communities. These communities exemplify collaborative efforts aimed at addressing societal challenges, where in‐group identification plays a crucial role (e.g. Bauwens, 2016). Specifically, studies have shown that residents who identify more strongly with their community in which the initiative is based are more willing to participate in that initiative (Goedkoop, Sloot, et al., 2022; Heath et al., 2017; Obst & White, 2007). Considering the strong relationship between in‐group identification and collaboration, it is essential to examine the underlying factors that determine in‐group identification within groups.

A key determinant of in‐group identification is interpersonal contact, which is essentially the direct interaction between group members (Pettigrew, 1998). Drawing on literature around in‐group identification and group perceptions (Blanchard et al., 2020; Campbell, 1958; Jans et al., 2011; Lickel et al., 2000; Postmes et al., 2005), greater frequency and quality of interpersonal contact among group members have been shown to be critical factors influencing the degree to which individuals identify with a group. This occurs because interactions among group members foster positive perceptions and experiences of being part of a group and strengthen emotional bonds (Jans et al., 2015; Leach et al., 2008). This leads us to expect that higher levels of interpersonal contact with group members predict stronger in‐group identification, in turn promoting collaboration intentions.

Building on this, spatial characteristics of a group setting may shape opportunities for interpersonal contact. Being located closer to one another may enhance such contact. The following section will explore the extent to which the spatial properties of a group setting may influence the frequency and nature of interactions among its members and, consequently, affect identification with a group.

### The influence of a group's spatial characteristics

Psychological literature on community energy initiatives has rarely considered how spatial distance influences key psychological factors. This is partly because research on psychological variables such as identification in energy initiatives has concentrated mostly on local contexts (Boon & Dieperink, 2014; Goedkoop, Dikstra, et al., 2022; Heath et al., 2017; Jans et al., 2024; Nientimp et al., 2024; Obst & White, 2007; Petersen, 2016; Sloot et al., 2018, 2019; Süsser et al., 2017). Beyond this research gap, there is also a common assumption in both research and policy that community initiatives are best suited to the local level. Yet, energy community collaboration can also emerge in broader, geographically dispersed group settings (Held & Corcoran, 2026), raising questions about how identification operates across spatial distance.

In social geography, for instance, research has demonstrated that spatial properties of groups shape social interactions, with greater physical distance between group members decreasing the likelihood of collaboration (Jessop et al., 2008; Massey, 2005). In a similar vein, sociological studies have highlighted how spatial factors influence network formation, with increased distance reducing the probability of social ties between people developing (Doreian & Conti, 2012; Felder et al., 2023; Hipp & Perrin, 2009; Nientimp et al., 2024; Small & Adler, 2019). For example, Hipp and Perrin (2009) found that spatial proximity affects the strength of social connections among neighbours, with those living closer forming stronger ties than those further apart. This underscores the significance of greater spatial distance (or, in this case, lesser proximity) from other group members in limiting the ability for people to interact and connect with one another.

A growing body of work has examined the more contextual, spatial elements, including community scale and the role that place plays in the formation of and participation in community energy initiatives (De Franco et al., 2023; Süsser et al., 2017). In addition, a systematic review by De Simone et al. (2025) identified community cohesion and local embeddedness as crucial indicators of participation in community energy initiatives. While these factors are not strictly spatial, they demonstrate how people's connection to place influences participation in such initiatives. Overall, the aforementioned collection of research has shown how ties and relations to people and place shape engagement with community energy initiatives and provide an important consideration for the present study.

Psychological studies specifically are yet to address how both actual distance and perceptions of distance from group members may influence collaboration intentions. As such, we build on the social identity perspective in examining how (perceived) spatial characteristics influence both interpersonal contact and the formation of group identification, further enhancing our understanding of socio‐spatial dynamics on community energy collaboration intentions.

We propose that greater (perceived) spatial distance from other group members will negatively affect their intentions to collaborate. This is grounded in the idea that increased (perceived) spatial distance from other group members reduces opportunities for interpersonal contact, which is essential for stimulating identification, and in turn collaboration intentions (Goedkoop, Sloot, et al., 2022; Thomas et al., 2016). Accordingly, we put forward the following hypotheses:

Hypothesis 1a Greater (perceived) spatial distance from group members will be negatively associated with individuals' levels of interpersonal contact with that group.

Hypothesis 1b Greater (perceived) spatial distance from group members will be negatively associated with individuals' levels of in‐group identification with that group.

Hypothesis 2 Higher levels of interpersonal contact will be positively associated with individuals' levels of in‐group identification with that group.

Hypothesis 3 Greater (perceived) spatial distance from group members will be associated with lower in‐group identification through lower interpersonal contact.

Hypothesis 4 Higher levels of in‐group identification will be positively associated with collaboration intentions with group members.

Hypothesis 5 Greater (perceived) spatial distance from group members will be associated with lower collaboration intentions through first lowering interpersonal contact and then lowering in‐group identification.

### Current research

We conducted two studies to test our reasoning. In both studies, we aimed to examine the underlying relationship between (perceived) spatial distance, interpersonal contact and in‐group identification. Study 1 tested the idea that distance had a negative relationship with in‐group identification (Hypotheses 1a and 1b) and that this relationship was mediated by interpersonal contact (Hypotheses 2 and 3). Study 2 retested Hypotheses 1a, 1b, 2 and 3 and further examined how all of this in turn predicts intentions to collaborate (Hypotheses 4 and 5).

## STUDY 1

In Study 1, we examined the relationship between in‐group identification and (perceived) distance from other group members, as well as the extent to which interpersonal contact mediated this relationship. We compared three groups that differed in spatial scale: neighbourhood, city and country. We did this because the average distance between an individual and group members is larger the larger the scale of the group is. Accordingly, this allowed us to examine how actual spatial distance aligns with perceived distance, interpersonal contact and in‐group identification, along with testing whether the hypothesised relations between perceived distance, interpersonal contact and in‐group identification operate across different spatial scales.

### Method

#### Participants and design

The study included a total of 195 participants. Our sample comprised first‐year students at a Dutch university who participated to obtain course credits, and a convenience sample obtained via the personal network of a member of the research team. There were no exclusions from the sample. Of the participants, 69.7% identified as female, 27.2% as male and the remaining 3% either identified as ‘other’ or did not disclose their gender. Participants' ages ranged from 18 to 69 years (*M* = 24.1, *SD* = 10.04). The study utilised a within‐subjects experimental design, so that each participant was exposed to all three spatial scales. The order in which these spatial scales were presented to participants was counterbalanced, either progressing from the most local group to the largest (neighbourhood to city to country) or reversed.

Prior to data collection, power analyses were conducted in G*Power (version 3.1; Faul et al., 2009) to estimate the required sample sizes for the planned tests. For the repeated measures ANOVA (fixed effects, omnibus), a medium effect size (*f* = 0.25; Cohen, 2016), an alpha level of .05 and desired power of .80 indicated that at least 159 participants would be needed to detect group differences. For the two‐tailed Pearson correlation, assuming a medium expected effect (*r* = .30; Cohen, 2016) under the same significance and power criteria, the analysis suggested a minimum of 84 participants. With 195 participants, the study surpassed both requirements and achieved sufficient statistical power.

#### Procedure

The following study was approved by the Ethics Committee at the University of Groningen, Faculty of Behavioural and Social Sciences. All participants were provided a link to an online survey hosted on Qualtrics. Upon accessing the survey, they received detailed information about the nature of the study and were asked to provide informed consent. Participants who consented proceeded with the survey, while those who did not were automatically redirected to the survey's end, thanked for their time and excluded from the data.

Participants were asked the same set of questions for their neighbourhood, city, and country. For each spatial scale, they were asked to report the extent to which they identified with and interacted with members of that group, as well as their perceptions of distance to each group. In addition to the core questions, several further items were included for purposes beyond the scope of this study and are therefore not reported here. Demographic information was collected at the end of the survey.

#### Measures

All measures were answered on a 7‐point Likert scale ranging from (1) *completely disagree* to (7) *completely agree*.

***In‐group identification*** with each group was measured using the following statement: ‘I identify with my neighbourhood (city; country)’ (Postmes et al., 2013).

***Interpersonal contact*** with group members was measured with the following statement: ‘I spend time interacting with most people of my neighbourhood (city; country)’ (Blanchard et al., 2020).

***Perceived Distance*** was measured with the question ‘I feel that residents of my neighbourhood (city; country) are distant from myself’ (Keller et al., 2022).

### Results

#### Testing differences between the groups

We first examined whether perceived distance from other group members differed across the three spatial scales. A repeated‐measures ANOVA revealed a significant within‐subjects effect of condition, *F* (2, 380) = 7.448, *p* < .001, *η* ^2^ *p* = .038. Pairwise comparisons with Bonferroni correction (see Table 1) showed that, unexpectedly, perceived distance in the neighbourhood condition was significantly higher than in the city condition, 95% CI [.173, .748], *p* < .001, but did not differ significantly from the country condition, *p* = .369, 95% CI [−.105, .482]. Perceived distance in the city and country conditions did not differ significantly, 95% CI [−.016, .561], *p* = .071.

**TABLE 1:** Mean and standard deviations of tested variables across spatial scales.

	Neighbourhood	City	Country
Perceived distance	4.13_*a*_ (1.46)	3.67_*b*_ (1.38)	3.92_*ab*_ (1.33)
Interpersonal contact	3.06_*a*_ (1.63)	3.81_*b*_ (1.61)	3.85_*b*_ (1.74)
In‐group identification	3.52_*a*_ (1.61)	4.30_*b*_ (1.52)	4.14_*b*_ (1.53)

*Note*: Means with different subscripts are significantly different at *p* < .05, Bonferroni corrected.

Interpersonal contact also differed across the three spatial scales, *F* (2, 384) = 20.443, *p* < .001, *η* ^2^ *p* = .096. Interpersonal contact was significantly lower in the neighbourhood than in both city conditions, 95% CI [−1.123, −.411], *p* < .001 and the country condition, 95% CI [−1.192, −.404], *p* < .001. Interpersonal contact levels in city and country conditions did not differ significantly, 95% CI [−.290, .228], *p* = 1.000.

Finally, in‐group identification differed across the three spatial scales, *F* (2, 386) = 18.834, *p* < .001, *η* ^2^ *p* = .089. Neighbourhood identification was significantly lower than both city conditions, 95% CI [−1.112, −.486], *p* < .001 and country conditions, 95% CI [−.974, −.305], *p* < .001. City and country in‐group identification did not differ significantly, 95% CI [−.190, .510], *p* = .814. Thus, actual distance from other group members connected to the different spatial scales does not directly predict greater perceived distance, and counter to Hypotheses 1a and 1b, actual spatial distance does not directly predict less interpersonal contact and less identification.

#### The connection between (perceived) distance, interpersonal contact and in‐group identification

Following this, we conducted Pearson's correlation analyses (see Table 2) to test whether perceived distance would be negatively associated with individuals' levels of interpersonal contact (Hypothesis 1a) and in‐group identification (Hypothesis 1b). As well as whether interpersonal contact would be positively associated with in‐group identification (Hypothesis 2). For both identification and interpersonal contact, the results indicated a negative association with perceived distance across the groups, with the associations appearing strongest at the neighbourhood level, and weakest at the country level. Interpersonal contact was also positively associated with in‐group identification across the groups, again with the associations appearing strongest at the neighbourhood, and weakest at the country level. As such, these findings consistently demonstrate that perceptions of greater distance were associated with lower levels of both interpersonal contact (Hypothesis 1a) and in‐group identification (Hypothesis 1b) and that interpersonal contact and in‐group identification were related (Hypothesis 2) across all three contexts, with this pattern being most pronounced in the more proximate group, the neighbourhood.

**TABLE 2:** Correlation table.

	Neighbourhood		City		Country	
	Interpersonal contact	In‐group identification	Interpersonal contact	In‐group identification	Interpersonal contact	In‐group identification
Perceived distance	−.55**	−.57**	−.43**	−.49*	−.25**	−.41**
Interpersonal contact	–	.63**	–	.43**	–	.30**

* *p* < .05;

** *p* < .01;

#### Understanding in‐group identification

**FIGURE 1:**
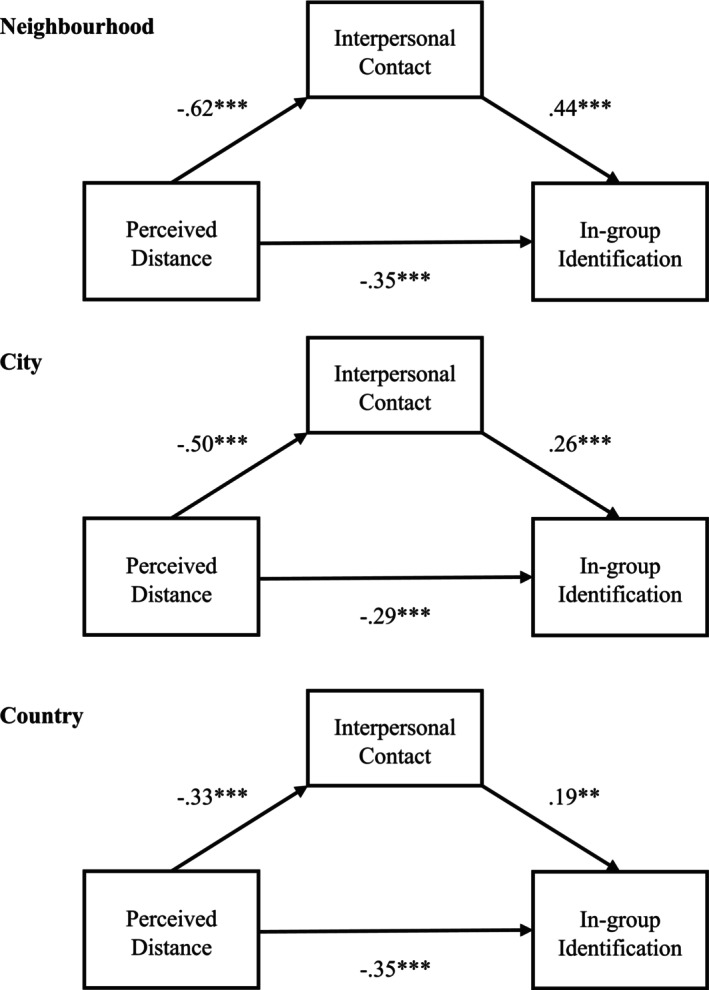
Mediation models predicting in‐group identification within each group. Path coefficients are standardised direct effects (β), with each path estimated controlling for all other paths in the model. **p < .01. ***p < .001.

We conducted a mediation analysis using Hayes' PROCESS macro (Model 4) to examine Hypothesis 3 within each group, which states that greater perceived distance from group members will be associated with lower in‐group identification through reduced interpersonal contact. Model 4 tests a simple mediation pathway, estimating both the direct effect of the predictor on the outcome and the indirect effect through the proposed mediator (Figure 1). We estimated the 95% confidence intervals using 5000 bias‐corrected bootstrap samples.

For the neighbourhood, the confidence intervals for the indirect effect of distance on in‐group identification through interpersonal contact did not contain zero, *β* = −.27, 95% CI [−.37, −.18], but the direct effect from distance to in‐group identification remained significant, indicating partial mediation. Overall, the model explained 46% of the variance in in‐group identification, *F* (2, 191) = 81.96, *p* < .001.

At the city level, the confidence intervals for the indirect effect of distance on in‐group identification through interpersonal contact did not contain zero, *β* = −.13, 95% CI [−.22, −.05], but the direct effect from distance to in‐group identification remained significant, indicating a partial mediation. This model explained 30% of the variance in in‐group identification, *F* (2, 188) = 40.95, *p* < .001.

At the country level, the confidence intervals for the indirect effect of distance on in‐group identification through interpersonal contact did not contain zero, *β* = −.06, 95% CI [−.13, −.01], but the direct effect from distance to in‐group identification remained significant, indicating a partial mediation. This model explained 21% of the variance in in‐group identification, *F* (2, 190) = 24.53, *p* < .001.

Across all three groups, we found a partial mediation of interpersonal contact on the relationship between perceived distance and in‐group identification. This supports Hypothesis 3, showing that increased perceived distance is associated with lower levels of interpersonal contact, which in turn relates to in‐group identification while also being directly related to in‐group identification.

### Discussion

To summarise, whilst the outcomes of the within‐group analyses supported our hypotheses, we did not find the expected effect of actual spatial distance on perceived distance, nor on identification, nor on interpersonal contact across the groups. Specifically, the group at the most local and proximate scale (the neighbourhood) was perceived as the most distant, and significantly more distant than the city. Moreover, respondents had less interpersonal contact and identified less with the neighbourhood than with the other groups, suggesting that these factors are more closely linked to perceived distance than the actual spatial distance. Our perceived‐distance measure therefore reflected subjective perceptions rather than validated objective distance, and may partly capture psychological or relational closeness.

Although these unanticipated findings may mean that our assumptions about spatial scale and distance are incorrect, they can also be explained by the student sample we used. Specifically, students may have fewer ties to neighbours, which could explain why they feel spatially closer to broader scales such as the city and country with whom they have clearer connections.

Within the groups, supporting Hypothesis 1a and 1b, we found that the more distant people perceived other group members to be, the less they had contact with the group members and identified with them. Hypothesis 2 was also supported across the groups with positive associations between interpersonal contact and in‐group identification. Moreover, in line with Hypothesis 3, part of the association between perceived distance and in‐group identification was explained by interpersonal contact. This pattern was consistent across all three group types, suggesting that these relationships hold across different spatial scales.

## STUDY 2

Study 2 adopted a similar approach to Study 1 but incorporated several modifications. In addition to examining the relationship between interpersonal contact, in‐group identification and perceived spatial distance (Hypotheses 1a, 1b, 2 and 3), we included a measure of collaboration intentions to explore how these factors influence potential engagement in a community‐based energy initiative (Hypotheses 4 and 5). Furthermore, we introduced two pictorial measures to capture people's perceptions of spatial distance, as an alternative, more concrete and visual way of testing perceived distance (or proximity) from group members. One pictorial measure focused on perceived centrality (for a description of the measure, see Measures section), which reflects how central the respondent perceived oneself within the group. The more central an individual is located within the group, the smaller the average distance between them and other group members, indicating more spatial proximity. The other pictorial measure focused on density (for a description of the measure, see Measures section), which reflected how densely group members are perceived to be located around the respondent. The denser group members are perceived to be located around the respondent, the smaller the average distance between the respondent and group members, indicating more spatial proximity. These measures allowed us to better examine which spatial distance perceptions are key to interpersonal contact, identification and collaboration intentions.

While for Study 1 the scale of the group (neighbourhood, city, country) was confounded by group size (i.e. broader groups are also larger), Study 2 examined groups that are more similar in size but often differ in spatial composition: neighbourhood, workplace and extended family. Specifically, neighbours are per default proximate to where someone is living; colleagues may be proximate while at work, but also more distant from home when working remotely or at dispersed locations; and (extended) family members are often more spread; while within each group substantial variations may also occur, providing good contexts to test our hypothesised processes. Moreover, these three contexts were selected because they represent plausible settings for community energy initiatives. On top of this, we moved from a student sample in Study 1 to a general population sample in this study to increase the generalizability of our findings.

### Method

#### Participants and design

Participants for this study were recruited from a US general population sample using an online panel company, Amazon Mechanical Turk (MTurk). A total of 287 respondents participated, with 34.5% identifying as female, 65.2% as male and 0.3% indicating other. Participants' ages ranged from 22 to 96 years (*M* = 34.3, *SD* = 9.50).

Conditions were set via MTurk to ensure that all participants were residing in the USA and were of the age of 18 or above. To ensure that the data collected was of sufficient quality and to exclude potential ‘bot’ responses, two screening methods were implemented. First, a CAPTCHA question was included at the start of the survey to prevent automated bots from participating. Second, participants were given a unique code at the end of the survey, which they were required to submit to confirm they had completed the questionnaire themselves (as recommended by Chmielewski & Kucker, 2020 and Kennedy et al., 2020).

The study employed a within‐subjects experimental design, allowing every participant to evaluate all three groups (neighbourhood, workplace and extended family) in a random order. A‐priori sample size estimations were conducted in G*Power (version 3.1; Faul et al., 2009) for the planned analyses. For the one‐way ANOVA (fixed effects, omnibus), assuming a medium expected effect (*f* = 0.25; Cohen, 2016), a significance level of .05 and desired power of .80, the analysis indicated that at least 159 participants would be required to detect group differences. For the two‐tailed Pearson correlation, assuming a medium expected effect (*r* = .30; Cohen, 2016), a significance level of .05, and desired power of .80, the analysis indicated that at least 84 participants would be required to detect a significant correlation. With 287 participants, the study surpassed the required sample size for both analyses and achieved sufficient statistical power.

#### Procedure

The following study was approved by the Ethical Committee at the University of Groningen, Faculty of Behavioural and Social Sciences. Participants were recruited via MTurk and provided with a link that directed them to a Qualtrics survey. Upon accessing the survey, participants were informed about the nature of the research and asked to provide informed consent. If consent was given, participants would then carry on into the survey. If not given, participants would be directly sent to the end of the survey, thanked for their participation and not recorded.

In accordance with Study 1, the order in which participants received the three group types was randomised, and each participant was presented with questions about their neighbourhood, workplace, and extended family in a random sequence. For each group, participants responded to questions assessing in‐group identification, interpersonal contact, perceived density and perceived centrality.

Following these measures, participants were asked about their intentions to collaborate in community activities, including their willingness to actively participate, become a member, seek information, and invest resources in a community energy initiative. Finally, participants provided demographic information (e.g. gender, age) and entered their MTurk ID to receive compensation for their participation.

#### Measures

*In‐group identification* was measured with the same item as Study 1 and adapted to each group type: ‘I identify with members of my neighborhood (workplace, extended family)’ (Postmes et al., 2013). Answers were provided on a 7‐point Likert scale ranging from (1) *completely disagree* to (7) *completely agree*.

*Interpersonal contact* was measured using the following items: ‘We spend time interacting,’ ‘We communicate with each other’ and ‘We interact with each other often’ (Blanchard et al., 2020). It was made clear to participants throughout that the word ‘*We*’ in the question referred to both the participant themselves and the relevant group members (i.e. my neighbours and I) to better inform participants' answers. Answers were provided on a 7‐point Likert scale ranging from (1) *completely disagree* to (7) *completely agree*, and the mean of these items was taken as the score of interpersonal contact. Internal consistency of the interpersonal contact scale across the neighbourhood (*α* = .84), workplace (*α* = .77) and extended family (*α* = .80) were good.

*Perceived distance* to group members was measured with two constructs. Namely, the perceived centrality as well as the perceived density of group members of the self within the group. Pictorial scales were created to measure these constructs, as these pictorial scales are better able to capture people's spatial perceptions of groups than in Study 1. (See Appendices S1 and S2 for the respective diagrams.)

For perceived *Centrality*, participants were shown seven diagrams (Appendix S1), depicting different levels of self‐centrality, from most central (1) to least central (7). Each diagram contained a circle representing the spatial composition of the group, a red dot that represented the participant, along with multiple black dots that represented other group members (e.g. colleagues). Depending on the centrality of the self to the group, the red dot (representing the participant) would range from being situated in the centre of the circle (most central) to progressively moving towards and outside of the circle's boundary (least central). The black dots remained constant in each image. After an explanation of these diagrams, participants were asked to select the diagram (1–7) that best represented their perception of their own centrality within the group.

For perceived *Density*, participants were shown seven diagrams (Appendix S2), each representing different levels of group density, ranging from most dense (1) to least dense (7). In each diagram, a circle represented the spatial composition of the group, with a red dot in the centre symbolising the participant and multiple black dots representing other group members (e.g. colleagues). Depending on the density of the group, the black dots would range from being concentrated around the participant (most dense) to completely dispersed to the periphery of the circle (least dense). After an explanation of these diagrams, participants were asked to select the diagram (1–7) that best represented their perception of their group's density.

Both sets of items were reverse‐coded to correctly represent centrality and density, meaning that higher scores indicated higher levels of perceived density and centrality, respectively.

*Collaboration intentions* were measured using four items. Initially, participants were to imagine that their group members were to set up a community energy initiative which would involve the coming together of community members to collectively promote sustainability behaviours and develop projects to generate their own renewable energy (See Appendix S3 for the full text). Based on this, we asked participants to what extent they were likely to: ‘…actively participate in this initiative’, ‘…become a member of this initiative’, ‘…seek information about this initiative’, and ‘…invest money in this initiative’ for each group (i.e. neighbourhood, workplace and extended family). Answers were provided on a 7‐point Likert scale ranging from (1) *extremely unlikely* to (7) *extremely likely*. We used the mean of these items to calculate a collaboration intention score. Internal consistency of the collaboration intention scale was good for the neighbourhood (*α* = .82), workplace (*α* = .81) and extended family (*α* = .79).

### Results

#### Testing differences between the groups

To examine whether perceived density and centrality ratings differed across groups, a repeated‐measures ANOVA was conducted. The analysis revealed no significant effect of group on perceptions of density, *F* (2, 572) = 0.99, *p* = .37, *η* ^2^ *p* = .003, nor on centrality, *F* (2, 572) = 1.65, *p* = .19, *η* ^2^ *p* = .006. The means and standard deviations for density and centrality are presented below in Table 3.

**TABLE 3:** Mean and standard deviations of tested variables across groups.

	Neighbourhood	Workplace	Extended family
Centrality	4.91_a_ (1.39)	4.75_a_ (1.38)	4.82_a_ (1.52)
Density	5.52_a_ (0.91)	5.44_a_ (0.93)	5.44_a_ (0.95)
Interpersonal contact	5.15_a_ (1.20)	5.45_c_ (0.99)	5.29_b_ (1.12)
In‐group identification	5.16_a_ (1.23)	5.28_b_ (1.19)	5.40_c_ (1.24)
Collaboration intentions	5.36_a_ (1.06)	5.35_a_ (1.04)	5.36_a_ (1.04)

*Note*: Means with different subscripts are significantly different at *p* < .05, Bonferroni corrected.

Further repeated measures ANOVAs showed that interpersonal contact, *F* (1.87, 534.52) = 9.58, *p* < .001, *η* ^2^ = .032 and in‐group identification, *F* (1.90, 543.49) = 7.24, *p* < .001, *η* ^2^ = .025, differed significantly across the three groups. For collaboration intentions, no significant differences between the groups were observed, *F* (2, 572) = .018, *p* = .982, *η* ^2^ < .001 (See Table 3). Specifically, interpersonal contact was highest in the workplace, followed by extended family, then neighbourhood. The pattern for in‐group identification was different, with participants reporting the highest identification in the extended family, followed by workplace, then neighbourhood. This seems to suggest that perceived density, centrality, interpersonal contact, in‐group identification and collaboration intentions are not related in the hypothesised way, at least not when looking across groups. To further examine the relation between perceived distance (density and centrality) and interpersonal contact, identification and collaboration intentions, the following section will test these relationships within the groups.

#### Correlations between the relevant measures within each group

We used Pearson's correlation analyses (see Table 4) to assess whether greater perceived distance from group members would be negatively related to interpersonal contact (Hypothesis 1a) and in‐group identification (Hypothesis 1b), whether interpersonal contact was positively associated with in‐group identification (Hypothesis 2) and whether identification was positively related to collaboration intentions (Hypothesis 4).

**TABLE 4:** Correlation table across groups.

	Density	Interpersonal contact	In‐group identification	Collaboration intentions
Neighbourhood				
Centrality	.47**	.11	.15*	.04
Density	‐	.00	−.00	−.04
Interpersonal contact		‐	.71**	.47**
In‐group Identification			–	.53**
Workplace				
Centrality	.46**	.17**	.08	.00
Density	–	−.01	.08	.05
Interpersonal contact		‐	.60**	.41**
In‐group Identification			–	.45**
Extended Family				
Centrality	.61**	.28**	.25**	.11
Density	–	.27**	.25**	.13*
Interpersonal contact		–	.78**	.46**
In‐group Identification			–	.43**

* *p* < .05;

** *p* < .01;

The findings partially supported Hypotheses 1a and 1b, though with notable variation across groups. Specifically, in line with Hypothesis 1a, perceived centrality and interpersonal contact were significantly positively correlated for extended family and workplace, but (and different from Study 1) not for the neighbourhood. Perceived density only correlated with interpersonal contact for the extended family, but not for the other groups. In addition, in line with Hypothesis 1b, perceived centrality and in‐group identification were significantly positively correlated for the neighbourhood and extended family, but not for the workplace. Perceived density was also significantly positively correlated with in‐group identification for extended family, but not for the other groups. Following Hypothesis 2, interpersonal contact was found to be positively associated with in‐group identification across the groups. Furthermore, in line with Hypothesis 4, higher levels of in‐group identification were strongly positively associated with collaboration intentions with group members in all three groups (see Table 4).

Overall, these results suggest that the relationships between perceived distance and both interpersonal contact and in‐group identification are not uniform, while identification was strongly and positively associated with collaboration intentions.

#### From perceived distance to identification and collaboration intentions

**FIGURE 2:**
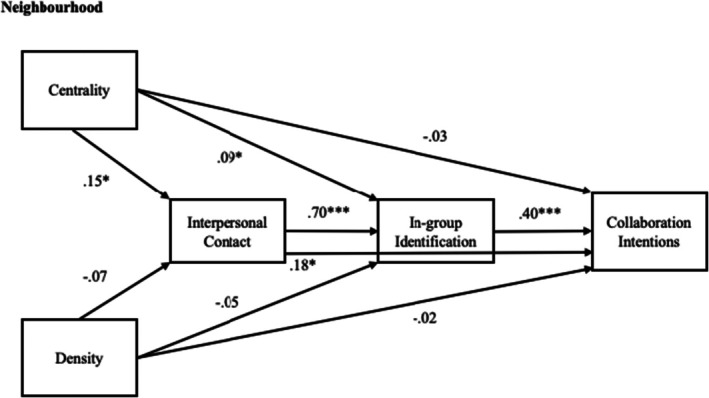
Serial Mediation Model, Predicting Neighbourhood Collaboration Intentions. Path coefficients are standardised direct effects (β), with each path estimated controlling for the mediators in the model. *p < .05, **p < .01, ***p < .001.

**FIGURE 3:**
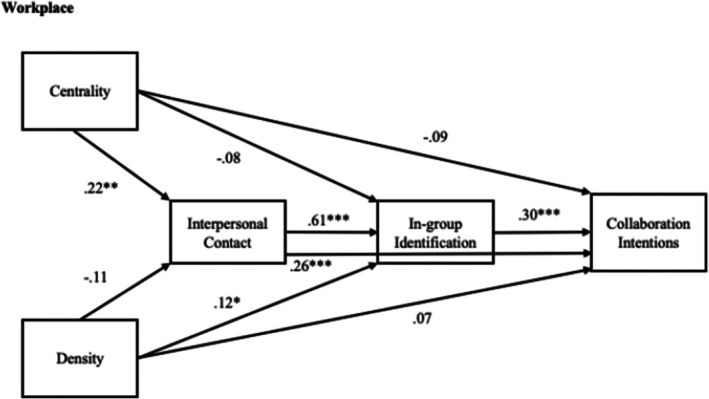
Serial Mediation Model, Predicting Workplace Collaboration Intentions. Path coefficients are standardised direct effects (β), with each path estimated controlling for the mediators in the model. *p < .05, **p < .01, ***p < .001.

**FIGURE 4:**
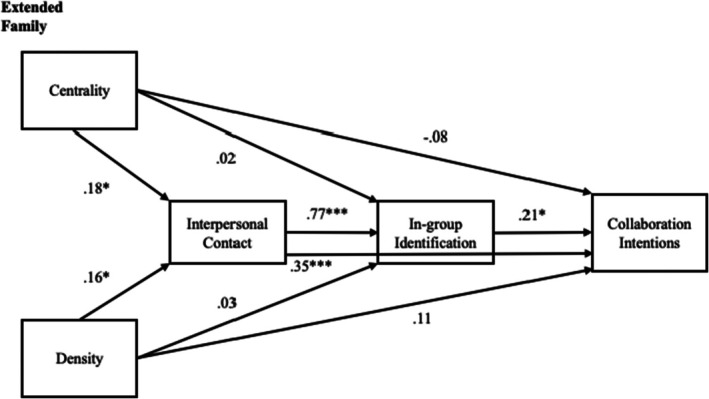
Serial mediation model, predicting extended family collaboration intentions. Path coefficients are standardised direct effects (β), with each path estimated controlling for the mediators in the model. *p < .05, **p < .01, ***p < .001.

To test Hypothesis 3 (distance → interpersonal contact → in‐group identification) and 5 (distance → interpersonal contact → in‐group identification → collaboration), we conducted a series of multiple regression analyses following a serial mediation approach. In Step 1, we regressed interpersonal contact (mediator 1) on density (predictor 1) and centrality (predictor 2). In Step 2, we regressed in‐group identification (mediator 2) on interpersonal contact, density and centrality. In the final Step 3, we regressed collaboration intentions (the outcome variable) on in‐group identification, interpersonal contact, density and centrality. The resulting coefficients are presented in Figure 2 for neighbourhoods, Figure 3 for workplaces and Figure 4 for extended families.

Where all associations of an indirect path were significant (e.g. centrality → interpersonal contact → in‐group identification → collaboration intentions), we tested this pathway further using Hayes' PROCESS macro. To examine Hypothesis 3, we used PROCESS's Model 4 at step 2 of the abovementioned multiple regression analysis (predictor: density or centrality, mediator: interpersonal contact; dependent variable: in‐group identification; covariate: centrality or density); to examine Hypothesis 5, we used PROCESS's Model 6 at step 3 of the abovementioned multiple regression analysis (predictor: density or centrality, mediator 1: interpersonal contact; mediator 2: in‐group identification; dependent variable: collaboration intentions; covariate: centrality or density). We estimated the 95% confidence intervals of the effect of each pathway using 5000 bias‐corrected bootstrap samples. VIF values were examined across all models to assess multicollinearity. Maximum VIF was 2.62, well below the recommended threshold of 5, indicating no multicollinearity concerns.

#### From distance to identification and collaboration intentions in the Neighbourhood

For the neighbourhood, the step 1 model explained 1.6% of the variance in interpersonal contact, *F* (2, 284) = 2.37, *p* = .095; the step 2 model explained 52% of the variance in in‐group identification, *F* (3, 283) = 100.62, *p* < .001; while the step 3 model explained 30% of the variance in collaboration intentions, *F* (4, 282) = 29.66, *p* < .001.

Focusing on Hypothesis 3, centrality but not density was positively related to interpersonal contact, and interpersonal contact was associated with in‐group identification at step 2 of our multiple regression analyses. Accordingly, we proceeded with only testing the hypothesised indirect path (distance → interpersonal contact → in‐group identification) for centrality. Despite the significant individual associations, the indirect effect was not significant, *β* = .09, 95% CI [−.02, .19], as the confidence intervals for the indirect effects contained zero. Hence, neither for density nor centrality do we find support for Hypothesis 3 that interpersonal contact mediates the relation between perceived distance and identification at the neighbourhood level.

Hypothesis 5 extends the path of Hypothesis 3 to collaboration intentions. Since Hypothesis 3 was already not supported, the sequential indirect effect from perceived distance (centrality or density) to interpersonal contact to in‐group identification to collaboration intentions will not be significant either. For this reason, we did not proceed with testing the full serial mediation of Hypothesis 5, despite all individual relationships of the path being significant for perceived centrality (see Figure 2). Accordingly, our data do not show support for Hypothesis 5 at the neighbourhood level.

#### From distance to identification and collaboration intentions at the workplace

For the workplace, the step 1 model explained 4% of the variance in interpersonal contact, *F* (2, 284) = 5.93, *p* < .01; the step 2 model explained 37% of the variance in in‐group identification, *F* (3, 283) = 54.40, *p* < .001; while the step 3 model explained 24% of the variance in collaboration intentions, *F* (4, 282) = 22.31, *p* < .001.

Regarding Hypothesis 3, centrality but not density was positively associated with interpersonal contact and interpersonal contact was related to in‐group identification at step 2 of our multiple regression analyses. As such, we proceeded with testing the hypothesised indirect path (distance → interpersonal contact → in‐group identification) for centrality. The indirect effect was significant, *β* = .12, 95% CI [.05, .19], as the confidence intervals for the indirect effects contained zero. The direct effect from centrality to in‐group identification was not significant (see Figure 3), suggesting that the relationship between centrality and in‐group identification operated indirectly through interpersonal contact in the workplace, in line with Hypothesis 3.

Hypothesis 5 extends the indirect path of Hypothesis 3 to collaboration intentions. Since in‐group identification was indeed positively associated with collaboration intentions, and Hypothesis 3 was only supported for perceived centrality, we proceeded with testing the full sequential path of Hypothesis 5 for perceived centrality. Here, the indirect effect of the sequential pathway from centrality to interpersonal contact to identification to collaboration intentions was also significant, *β* = .04 (95% CI [.01, .09]). As such, in the workplace we do find support for Hypothesis 5.

Inspections of other possible paths did indicate a significant indirect path from density to in‐group identification to collaboration, *β* = .07, 95% CI [.01, .12], suggesting that perceived density effects operate through in‐group identification.

#### From distance to identification and collaboration intentions in the extended family

For the extended family, the step 1 model explained 9% of the variance in interpersonal contact, *F* (2, 284) = 14.53, *p* < .001; the step 2 model explained 61% of the variance in in‐group identification, *F* (3, 283) = 148.74, *p* < .001; while the step 3 model explained 29% of the variance in collaboration intentions, *F* (4, 282) = 28.92, *p* < .001.

For Hypothesis 3, centrality and density were positively associated with interpersonal contact, and interpersonal contact was related to in‐group identification at step 2 of our multiple regression analyses. As such, we proceeded with testing the hypothesised indirect path (distance → interpersonal contact → in‐group identification) for both centrality and density. Interpersonal contact mediated the relationship between centrality and in‐group identification, *β* = .11, 95% CI [.02, .21]. The direct effect from centrality to in‐group identification became non‐significant in the mediation model (see Figure 4), suggesting that the relationship between centrality and in‐group identification was fully mediated by interpersonal contact. Similarly, interpersonal contact also fully mediated the relationship between density and in‐group identification, *β* = .16, 95% CI [.02, .29]. The direct effect of density on in‐group identification became non‐significant in the mediation model (see Figure 4). Hence, supporting Hypothesis 3 for both centrality and density in the extended family.

Hypothesis 5 extends the indirect path of Hypothesis 3 to collaboration intentions. We proceeded with testing the full sequential path of Hypothesis 5 for perceived centrality and density. For centrality, the sequential route was not significant, *β* = .03, 95% CI [−.0002, .08]. Thus, it does not support Hypothesis 5 in this case. For density, the indirect effects via the sequential pathway were also not significant, *β* = .02, 95% CI [−.002, .06].

Examination of other possible paths revealed an indirect effect of centrality on collaboration intentions through interpersonal contact, *β* = .10, 95% CI [.02, .17], as well as a significant indirect effect of density on collaboration through interpersonal contact, *β* = .05, 95% CI [.002, .13]. Suggesting that both perceived centrality and density effects can operate through interpersonal contact alone without the step through in‐group identification.

#### Discussion

To summarise, although we find support for our general proposition that greater perceived distance (indirectly) predicts lower identification and collaboration intentions, the exact process through which this happens seems to vary between groups and seems more consistent for perceived centrality than density. Specifically, we find evidence that greater perceived centrality is related to stronger identification, via increased interpersonal contact (in line with Hypotheses 1a, 1b and 3), in the workplace and extended family. Whereas the steps of the path were also significant in the neighbourhood, the indirect effect on in‐group identification was not. Instead, greater perceived centrality was solely directly related to stronger identification (only in line with Hypothesis 1b). Whilst in all groups there is a positive relation between interpersonal contact and in‐group identification (Hypothesis 2) and with identification and collaboration intentions (in line with Hypothesis 4), we only find full support for the sequential positive relation between perceived centrality and collaboration intentions via interpersonal contact and identification in the workplace (in line with Hypothesis 5). Interestingly, in the neighbourhood we observed a positive indirect association between perceived centrality and collaboration intentions via increased in‐group identification (only partially supporting Hypothesis 5, without interpersonal contact), while in the extended family we observed a positive indirect association between perceived centrality and collaboration intentions via increased interpersonal contact (only partially supporting Hypothesis 5, without in‐group identification).

For the relation between greater perceived density and stronger identification, via increased interpersonal contact, we only find support in the extended family (in line with Hypotheses 1a, 1b and 3). In turn, it seems that this increased interpersonal contact is what particularly explains the relation between stronger perceived density and collaboration intentions with this group (only partially supporting Hypothesis 5, without identification). Instead, in the workplace, greater perceived density is only directly related to stronger identification (only in line with Hypothesis 1b), and this identification in turn solely predicts stronger collaboration intentions in this group (only partially supporting Hypothesis 5, without interpersonal contact). Finally, in the neighbourhood, perceived density does not relate to interpersonal contact, identification, or collaboration intentions at all. Thus, whilst the results generally suggest that greater perceived distance (indirectly) predicts lower identification and collaboration intentions, perceived centrality appears particularly relevant in predicting collaboration intentions, either through interpersonal contact, identification or sequentially through both, depending on the specific characteristics of the group.

## GENERAL DISCUSSION

This research examined whether spatial characteristics of groups influence the social‐psychological processes that support intentions to collaborate in community energy initiatives. Across two studies, we tested whether spatial distance and perceived spatial distance shaped opportunities for interpersonal contact, in‐group identification and then collaboration intentions. Whereas actual spatial distance seemed unrelated to perceived distance and did not consistently affect interpersonal contact, in‐group identification or collaboration intentions, most of our hypotheses were to some degree supported by our regression analyses that used perceived distance as the predictor variable. That said, substantial variations occurred across operationalisations of perceived distance and group types.

Overall, the findings suggest that collaboration intentions depend less on the actual spatial composition of groups and more on perceptions of distance, which are linked to the interpersonal contact and/or identification processes already emphasised in social identity literature (Goedkoop, Sloot, et al., 2022; Heath et al., 2017; Obst & White, 2007; Rees & Bamberg, 2014; Sloot et al., 2018). Importantly, the associations we found appear more relevant for determining collaboration intentions within groups than for identifying suitable groups for collaboration. That is, which members are most likely to be willing to collaborate within a given group, rather than with which groups a given person is most willing to collaborate.

### Between‐group comparisons

Across both studies, we tested different groups that people may hold different perceptions of distance for. However, contrary to our expectation, the objectively most proximal group (i.e. the neighbourhood) was not perceived as the most proximate in our studies and was even regarded as the most distant in Study 1. In addition to not finding anticipated group differences in perceived distance, people also did not interact with, identify with or intend to collaborate more with spatially proximate groups compared to distant ones. In other words, differences in spatial scale between groups (e.g. neighbourhood, country, family) were poor predictors of these outcomes. Although these findings contradict our hypotheses that less actual distance will lead to higher levels of in‐group identification and collaboration intentions, they suggest an important implication that groups with different spatial compositions may be equally effective for fostering intentions to collaborate in energy communities.

These findings highlight that people's perceptions of a group's distance may diverge from its objective, spatial properties, which underscores the psychological significance of the subjective experience of distance over the actual spatial composition of the group. In this respect, our results suggest that distance may function less as a fixed spatial constraint, as often implied in geography and sociology literature (Jessop et al., 2008; Massey, 2005), and more as a psychological one (Hipp & Perrin, 2009; Wilson et al., 2008) that may shape how members identify with a group. Future research could further explore how perceived distance is construed in people's minds by explicitly differentiating between its spatial, emotional and social components.

### Within‐group dynamics

Whereas actual distance between groups did not predict collaboration intentions suitability, perceived distance within groups was associated with in‐group identification and collaboration intentions, often via interpersonal contact. Our first hypothesis examined whether smaller perceived distance from other group members would be associated with stronger interpersonal contact and in‐group identification, while our second hypothesis tested the relation between interpersonal contact and in‐group identification. Accordingly, our Hypothesis 3 tested the association between perceived distance and in‐group identification being explained through interpersonal contact.

In Study 1, we found consistent evidence that greater perceived distance was correlated to lower levels of interpersonal contact and identification, across groups ranging in spatial scale from the neighbourhood to the country. This suggests that the subjective sense of how close or distant group members feel to each other is linked to the degree of psychological connection individuals experience with their group. Moreover, across the groups, we found the association between perceived distance and identification was partly mediated by interpersonal contact. As anticipated, individuals who perceived the group as less distant indicated more frequent interaction with group members, which was associated with stronger identification.

Corroborating Study 1, Study 2 generally supports our proposition that greater perceived distance is (indirectly) related to lower identification, and, extending Study 1, also somewhat to collaboration intentions (Hypothesis 5). Yet, results are more mixed, and the exact process through which perceived distance, in‐group identification and collaboration intentions are related varies between measures of perceived distance (centrality vs. density) and across groups, on each of which we reflect below.

Regarding the measure of perceived distance, perceived centrality was consistently (indirectly) related to identification and collaboration intentions across all groups. Perceived density, however, was not related to any of these outcomes in the neighbourhood, and was weakly related to increased in‐group identification and collaboration intentions in the workplace and the extended family, in different ways. Hence, how central or remote an individual is positioned within the group (i.e. centrality), rather than how spatially dispersed the group members in general are from each other (i.e. density), seems to overall matter more for in‐group identification and collaboration intentions. This could indicate that in‐group identification and collaboration intentions may depend less on the (perceived) spatial properties of the group, as we also noted earlier when comparing group means, and more on how individuals uniquely experience and position themselves within the group.

Looking at the groups in more detail, the relation between perceived centrality and collaboration intentions appears to be particularly attributable to interpersonal contact in the extended family, more to identification in the neighbourhood, and to both interpersonal contact and identification in the expected sequential order in the workplace. In addition, the association between density and collaboration intentions went sequentially via interpersonal contact and identification for the extended family, via identification in the workplace and did not occur for the neighbourhood. These differing mechanisms between the groups suggest that the pathways to collaboration intentions are more diverse than our sequential model proposes, with effects sometimes being more direct and differing in size depending on the group studied.

These variations between processes within groups may themselves be explained by differences in the underlying spatial organisation of those groups. For instance, in the neighbourhood, density was not found to be in direct or indirect relation to collaboration intentions. Since neighbourhoods are inherently spatially defined, whereby definition everybody is relatively proximate and density variations may be small, the variance in density may be insufficient to shape collaboration intention outcomes. In contrast, extended family and workplace settings may require a denser distribution of members to be linked with collaboration intentions, precisely because density is not always a given in these contexts. Colleagues may be dispersed, especially outside work hours and family members are often split across different locations.

Next to these differences in spatial organisation, the variation in observed processes may also arise from the psychological function and foundation of the studied groups. For instance, the Study 2 groups’ neighbourhood, workplace and (extended) family respectively map onto the group types, social category, task group and intimacy group identified by Lickel et al. (2000), each having their own dynamics and processes that lead to identification and collaboration intentions. One could, for instance, assume that identification with neighbours strongly depends on interpersonal contact, simply because apart from place there is little that bonds neighbours together, and that such identification is essential for neighbours' willingness to collaborate with each other. Conversely, the opposite may apply to (extended) family, where interpersonal contact may rather be the product of identification (e.g. based on genetic and shared history) and is more directly linked to collaboration intentions, possibly also for the practical reason that collaborations only make sense when interacting frequently enough. Obviously, these explanations are post hoc and speculative, and many different relevant classifications of group types exist (e.g. dynamic vs. categorical groups, Wilder & Simon 1998, interpersonal networks vs. social categories, Deaux & Martin, 2003; common identity vs. common bond groups, Prentice et al., 1994; see also the distinction between inductive and deductive processes of social identity formation; Postmes et al., 2005), stressing the need for further research on this.

It is important to note that while operationalisations of (perceived) distance seem to relate differently to in‐group identification and collaboration intentions across groups, interpersonal contact, in‐group identification and collaboration intentions were strongly associated with each other throughout. In‐group identification was consistently positively associated with collaboration intentions across the group contexts in Study 2, consistent with social identity theory, which links in‐group identification to collaborative behaviours (Haslam & Ellemers, 2011; Tajfel et al., 1979) and confirming previous research demonstrating its role in fostering community energy engagement (e.g. Bauwens, 2016). In addition, and in line with our theorising, interpersonal contact was related both directly to collaboration intentions and indirectly through its role in strengthening in‐group identification, supporting earlier work that highlights interpersonal interaction as a plausible basis for in‐group identification (Jans et al., 2015; Pettigrew, 1998) and collaboration (Goedkoop, Sloot, et al., 2022; Thomas et al., 2016).

### Limitations

The present studies are subject to certain limitations that should be acknowledged. One relates to the use of a student sample in Study 1. As mentioned, participants rated their neighbourhood as more distant compared to the city and country levels. This unexpected pattern may be due to the characteristics of the sample, in that students are often more mobile, have lived in their current neighbourhoods for only a short time, and may have weaker ties to local communities. As a result, they may feel less connected to their neighbourhood than to broader reference groups such as the city or country. While we were still able to test our proposed model within each group context, it may not have fully captured how the general population perceives distance across different group settings. In addition, the differential effects of distance across the two studies may also reflect the sample differences, with the student sample's past mobility and weaker neighbourhood ties compared to a general population sample. Future research should therefore systematically investigate how perceptions of distance and spatial properties influence collaboration across diverse populations and group contexts to determine whether these findings generalise beyond the specific samples examined here.

Furthermore, it should be noted that the perceived distance measures used in this paper have not been validated yet. Study 1's perceived‐distance measure was found to be unrelated to the objective spatial scale and may therefore reflect psychological or relational closeness rather than sole spatial closeness. The novel pictorial measures in Study 2 helped to distinguish different spatial indicators further; yet, they should be treated as exploratory indicators and will require further validation going forward. Future research should therefore further examine the relation between objective and subjective perceptions of distance and the validity of different perceived distance indicators.

Furthermore, it is important to note that the second study measures intentions towards joining and participating in a hypothetical community energy initiative. While intentions serve as an established and important precursor to actual behaviour, the gap between intention and behaviour (Sheeran, 2002) may result in an overestimation of actual collaboration rates in real‐world settings. To strengthen confidence in the proposed model and its practical applicability, future research should test these findings within the context of existing community energy initiatives, comparing whether the mechanisms of identification and distance operate similarly for both established members and potential newcomers considering participation. This will also help to bridge the gap between our novel perspective on the relation of perceived distance and current psychological literature on community energy collaboration.

Finally, the serial mediation models in Study 2 only explain a small amount of the variance in explaining collaboration intentions. This may be due to the fact of focusing on only one proposed sequential pathway from perceived distance, through interpersonal contact and then through identification, and did not consider other factors in our studies. Energy community literature, however, outlines many other individual and group‐level factors that explain participation, such as different forms of trust, environmental values and collective efficacy (De Simone et al., 2025). All these aspects may also be associated with collaboration intentions and therefore should also be integrated into a more substantial model in future research.

### Practical implications

Our findings carry certain considerations for how energy community initiatives may be conceptualised in policy and practice. Literature and practice often assume that such initiatives are best suited to the local level as physical proximity is thought to facilitate community collaboration. However, our results suggest a more nuanced direction, in that actual distance between members seems less important than the subjective sense of that distance. Specifically, the first study demonstrated that physically proximal groups are not always necessarily the ones in which members feel most connected, given that the neighbourhood condition was not the spatial scale to which student participants felt closest. This challenges the assumption that local settings are universally the best fit for community energy initiatives. For policymakers, it may be worth acknowledging that community initiatives based on certain locations or geographical boundaries may only be successful to the extent that people feel close. Rather than defaulting to neighbourhood‐level organisation, policy should consider whether the chosen geographical boundaries contain the sense of community identity and interpersonal contact that may be necessary in fostering collaboration.

Our findings demonstrate the plausible role of centrality in strengthening in‐group identification, suggesting that empowering local leaders to foster feelings of centrality within communities is one recommendation for promoting wider participation intentions. Initiators may target those who feel relatively central within a group or even prioritise creating opportunities for members to experience centrality, for instance by giving people more active roles within the community to stimulate such perceptions.

Our research suggests the relevance of emphasising the communal and group dimension when promoting collaboration intentions in energy‐related initiatives. A growing body of literature shows the connection that in‐group identification has with collaborative behaviours, particularly in addressing complex societal challenges like climate change (Fritsche et al., 2018). Importantly, our findings suggest that fostering in‐group identification may stimulate collaboration intentions within a given group.

In addition to fostering identification, we also underline the potential role of interpersonal contact in stimulating collaboration intentions. Research consistently shows that frequent and meaningful interaction among group members strengthens in‐group identification and enhances community collaboration behaviours (Blanchard et al., 2020; Campbell, 1958; Jans et al., 2015; Lickel et al., 2000; Postmes et al., 2005). For energy community initiators and leaders, facilitating opportunities and spaces for such interactions, whether through organised events or shared communal spaces, may deepen members' sense of belonging and reinforce their commitment to group goals (Blanchard & Markus, 2004).

By prioritising opportunities for meaningful interaction and identification alongside strategic engagement with community leaders, community energy initiatives may not need to be restricted to local settings. More diverse and geographically dispersed groups may also provide a solid foundation for community engagement in the energy transition, provided that these opportunities for connection are intentionally designed and sustained.

## CONCLUSION

This research strives to provide a novel contribution by examining the role of distance and perceived distance on collaboration intentions within community energy. Our key finding is that perceived distance indirectly shaped collaboration intentions within certain group contexts, whereas actual distance showed no relationship to collaboration intentions. As expected, collaboration intentions were most strongly predicted by interpersonal contact and identification, indicating that distance matters only insofar as it influences these underlying processes. Our results suggest that community initiatives may not be constrained by the actual distance of group members, but rather by their subjective experience of distance. Success could depend on creating opportunities for interaction and fostering group identification, both of which can be developed even in larger, more spatially dispersed settings. Ultimately, fostering these processes may prove key not only to community energy but to effective collaboration in pursuit of broader societal goals.

## AUTHOR CONTRIBUTIONS

**Charlie J. Walker Clarke:** Conceptualisation; methodology; formal analysis; investigation; visualisation; writing – original draft. **Elliot J. Sharpe:** Supervision; conceptualisation; methodology; writing – review and editing. **Lise Jans:** Conceptualisation; writing – review and editing. **Thijs Bouman:** Funding acquisition; project administration; supervision; conceptualisation; methodology; writing – review and editing.

## CONFLICT OF INTEREST STATEMENT

The authors declare that there are no conflicts of interest with respect to the research, authorship and/or publication of this article.

## Supporting information


**Appendix S1.** Questionnaire item where participants indicated their perceived centrality within the group for each group setting.
**Appendix S2.** Questionnaire item where participants indicated the perceived group density for each group setting.
**Appendix S3.** Questionnaire item that presented the hypothetical initiative to participants, where they went on to indicate their collaboration intentions.

## Data Availability

The data that support the findings of this study are available from the corresponding author upon reasonable request.
