# Supplementary material for: Beyond proximity: The social and spatial dynamics of collaboration in community energy initiatives
Source: Br J Soc Psychol. 2026 Aug 7;65(4):e70121. doi: 10.1111/bjso.70121 (PMC13448857; doi:10.1111/bjso.70121)
Supplement: Supplementary file 1 [file BJSO-65-0-s001.docx]

**Appendix**

Appendix S1 - Questionnaire item where participants indicated their perceived centrality within the group for each group setting.

Appendix S2 - Questionnaire item where participants indicated the perceived group density for each group setting.

Appendix S3 - Questionnaire item that presented the hypothetical initiative to participants, where they went on to indicate their collaboration intentions.
